# The efficacy and safety of asleep and awake subthalamic deep brain stimulation for Parkinson’s disease patients: A 1-year follow-up

**DOI:** 10.3389/fnagi.2023.1120468

**Published:** 2023-04-18

**Authors:** Wanru Chen, Changming Zhang, Nan Jiang, Lulu Jiang, Qiyu Guo, Jing Gu, Wenbiao Xian, Yuting Ling, Yanmei Liu, Yifan Zheng, Lei Wu, Chao Yang, Shaohua Xu, Yu Hu, Yang Yang, Jinhua Chen, Ruoheng Xuan, Yi Liu, Jinlong Liu, Ling Chen

**Affiliations:** ^1^Department of Neurology, The First Affiliated Hospital, Sun Yat-sen University, Guangdong Provincial Key Laboratory of Diagnosis and Treatment of Major Neurological Diseases, National Key Clinical Department and Key Discipline of Neurology, Guangzhou, China; ^2^Department of Neurology, Sun Yat-sen Memorial Hospital, Sun Yat-sen University, Guangzhou, China; ^3^Department of Neurosurgery, The First Affiliated Hospital of Sun Yat-sen University, Guangzhou, China; ^4^Department of Anesthesiology, First Affiliated Hospital of Sun Yat-sen University, Guangzhou, Guangdong, China; ^5^Department of Neurology, The First People’s Hospital of Huizhou City, Huizhou, Guangdong, China; ^6^Department of Medical Statistics and Epidemiology, School of Public Health, Sun Yat-sen University, Guangzhou, China; ^7^The East Division of the First Affiliated Hospital, Sun Yat-sen University, Guangzhou, China

**Keywords:** Parkinson disease, deep brain stimulation (DBS), general anesthesia (GA), local anesthesia (LA), follow-up, asleep DBS

## Abstract

**Introduction:**

Traditional DBS is usually conducted under local anesthesia (LA) which is intolerable to some patients, DBS under general anesthesia (GA) was opted to extended surgical indication. This study aimed to compare the efficacy and safety of bilateral subthalamic deep brain stimulation (STN-DBS) for Parkinson’s disease (PD) under asleep and awake anesthesia state in 1-year postoperative follow-up.

**Methods:**

Twenty-one PD patients were assigned to asleep group and 25 patients to awake group. Patients received bilateral STN-DBS under different anesthesia state. The PD participants were interviewed and assessed preoperatively and at 1-year postoperative follow-up.

**Results:**

At 1-year follow-up, compared surgical coordinate in two groups, the left-side Y of asleep group showed more posterior than awake group (Y was-2.39 ± 0.23 in asleep group, −1.46 ± 0.22 in awake group, *p* = 0.007). Compared with preoperative OFF MED state, MDS-UPDRS III scores in OFF MED/OFF STIM state remained unchanged, while in OFF MED/ON STIM state were significantly improved in awake and asleep groups, yet without significant difference. Compared with preoperative ON MED state, MDS-UPDRS III scores in ON MED/OFF STIM, and ON MED/ON STIM state remained unchanged in both groups. In non-motor outcomes, PSQI, HAMD, and HAMA score significantly improved in asleep group compared to awake group at 1-year follow-up (PSQI, HAMD, and HAMA score in 1-year follow-up were 9.81 ± 4.43; 10.00 ± 5.80; 5.71 ± 4.75 in awake group, 6.64 ± 4.14; 5.32 ± 3.78; 3.76 ± 3.87 in asleep group, *p* = 0.009; 0.008; 0.015, respectively), while there was no significant difference in PDQ-39, NMSS, ESS, PDSS score, and cognitive function. Anesthesia methods was significantly associated with improvement of HAMA and HAMD score (*p* = 0.029; 0.002, respectively). No difference in LEDD, stimulation parameters and adverse events was observed between two groups.

**Discussion:**

Asleep STN-DBS may be considered a good alternative method for PD patients. It is largely consistent with awake STN-DBS in motor symptoms and safety. Yet, it showed higher improvement in terms of mood and sleep compared to awake group at 1-year follow-up.

## 1. Introduction

Parkinson’s disease (PD) is one of the most disabling chronic neurologic diseases that significantly affects life quality (Nutt and Wooten, 2005; Li et al., 2019). Standard medication therapy can be used to alleviate PD symptoms. However, long-term medical management is often complicated with the appearance of levodopa-induced motor complications (Nutt and Wooten, 2005; Weaver et al., 2009). Subthalamic deep brain stimulation (STN-DBS) has been shown to be superior to standard medication therapy alone in improving motor function and quality of life in advanced PD (Liang et al., 2006; Gervais-Bernard et al., 2009; Weaver et al., 2009).

Traditional DBS is usually conducted under local anesthesia (LA). Yet, some patients who undergo LA may experience anxiety, respiratory distress, and uncontrolled hypertension (Collins and Wanklyn, 2002; Lin et al., 2008). Thus, several centers have opted for DBS under general anesthesia (GA) or asleep state to solve this problem (Foley, 2009; Maldonado et al., 2009). So far, several clinical trials of deep brain stimulation have been performed to compare efficacy and safety of awake and asleep DBS. In terms of motor symptoms, in comparison of 6 months to 1 year after DBS, most studies reported no difference in motor symptoms improvement between the two methods (Foley, 2009; Chen et al., 2011, 2018; Kwon et al., 2016; Lefranc et al., 2017; Blasberg et al., 2018; Tsai et al., 2019; Holewijn et al., 2021). By contrast, Blasberg reported that axial symptoms such as dysarthria and freezing in asleep group deteriorate with time compared to those in awake group (Blasberg et al., 2018). Previous domestic and international clinical studies have mostly focused on motor symptoms, while investigation of non-motor symptoms is relatively brief.

In terms of non-motor symptoms, most studies showed that two groups resulted in comparable improvement in quality of life, drug consumption reduction, and mood (Collins and Wanklyn, 2002; Kwon et al., 2016; Chen et al., 2018; Tsai et al., 2019). In contrast, Brodsky reported a better improvement in quality of life and speech fluency in asleep group than in awake group at 6 months after DBS (Foley, 2009). Moreover, Chen identified a significant deterioration in asleep group’s cognitive function compared to awake group after 1-year follow-up (Chen et al., 2011). Lefranc and colleagues reported that Levodopa equivalent daily dose (LEDD) reduction in asleep group was higher than that in awake group at 1-year follow-up (Lefranc et al., 2017).

Difference in efficacy and safety between awake and asleep STN-DBS surgery still remains ambiguous and needs more clinical reports (Sheshadri et al., 2017). Therefore, we conducted a comprehensive follow-up study to compare 1-year postoperative results from consecutive patients who underwent asleep STN-DBS with those who underwent the procedure under awake state. In this report, we assessed efficacy and safety of two methods by comparing stereotactic coordinates, motor and non-motor outcomes, medication dose, stimulation parameters, and adverse events.

## 2. Materials and methods

### 2.1. Patient selection and preoperative evaluation

A total of 53 consecutive PD patients who underwent bilateral STN-DBS at The First Affiliated Hospital of Sun Yat-sen University between August 2013 and June 2018 were enrolled in this study. The choice of anesthesia was based on time period. Twenty-four patients received bilateral STN-DBS electrode implantation under LA state between August 2013 and March 2016. Twenty-nine patients received bilateral STN-DBS electrode implantation under GA state between April 2016 and June 2018. Among 53 patients, we collected all patients’ adverse effects, but only 46 completed the whole evaluation. Three patients in awake group were excluded from this study; two cases were lost to follow-up, and one case committed suicide (she had very low emotional symptoms). Four patients in asleep group were excluded; one complicated with neuromuscular disorders and three cases were lost to follow-up.

Inclusion criteria were following (Jiang et al., 2015): PD diagnosis that met British Parkinson’s Disease Society Brain Bank criteria; Course of the disease >5 years; levodopa-induced motor complications, including dyskinesia or end-dose phenomena, occurred under the optimal treatment regimen of anti-Parkinson’s disease drugs; at least 30% levodopa response improvement of Movement Disorder Society-Unified Parkinson’s Disease Rating Scale Part III (MDS-UPDRS III) in levodopa challenge test; 18–75 years old; Magnetic resonance imaging (MRI) of head is normal; emotion and intelligence are normal, and MMSE scale >26 points; signed informed consent. Exclusion criteria were (Jiang et al., 2015): secondary Parkinson’s syndrome or multiple system degeneration; existence of serious metabolic disease, organ disease or mental illness; were not able to complete follow-up; other problems, such as low education, language barriers, and poor compliance; pregnant or lactating women. The same team, including neurosurgery, neurologist, and anesthesiologist, monitored and performed all of the DBS procedures in these patients for high homogeneity at our hospital.

Before the patient underwent surgery, he/she was observed for 1–2 months, after which the patient’s baseline condition was evaluated. During this period, the patient’s medication regimen remained the same without adjustment. Preoperative evaluation included assessment of motor symptoms and non-motor symptoms. MDS-UPDRS III was used to evaluate patient’s motor symptoms; main symptoms were divided into following categories: bradykinesia (items 4a-8b, 14), tremor (items 15a–18), rigidity (items 3a–3e), and axial score (item 1, 2, 9–13). The levodopa challenge test was performed about a week before surgery. The patient’s “OFF MED” state (the worst state before taking medicine) and “ON MED” state (the best state after taking medicine) were recorded. Before the test, all anti-Parkinson’s drugs were temporarily suspended; dopamine receptor agonists were stopped at least 72 h, and levodopa 12 h before testing. When “OFF MED” state evaluation was completed after drug discontinuation, the patient was instructed to chew a certain amount of levodopa (Levodopa dose = normal dose of medication in the morning × 150%, to avoid vomiting caused by large doses of drugs, taking Domperidone 10–30 mg half an hour in advance). The “ON MED” state evaluation was completed after observing the gradual improvement of motor symptoms and reaching the optimal state. The progress generally took about 4 ~ 6 h.

Among non-motor function assessment, patients were evaluated in several aspects. Sleep function was evaluated by Parkinson’s Disease Sleep Scale Chinese Version (PDSS-CV), Epworth Sleepiness Scale (ESS), and Pittsburgh Sleep Quality Index (PSQI). Emotion was evaluated by Hamilton Anxiety Scale (HAMA), Hamilton Depression Scale (HAMD), and Beck Depression Inventory (BDI). Cognitive function was assessed by Montreal Cognitive Assessment (MoCA) and Mini-mental State Examination (MMSE). Non-Motor Symptoms Scale (NMSS) was used to evaluate the overall non-motor symptoms. Quality of life was assessed by Parkinson’s Disease Questionnaire-39 (PDQ-39). Levodopa equivalent daily dose was recorded.

### 2.2. Surgery and anesthesia procedure

#### 2.2.1. Imaging and targeting

**Figure 1:**
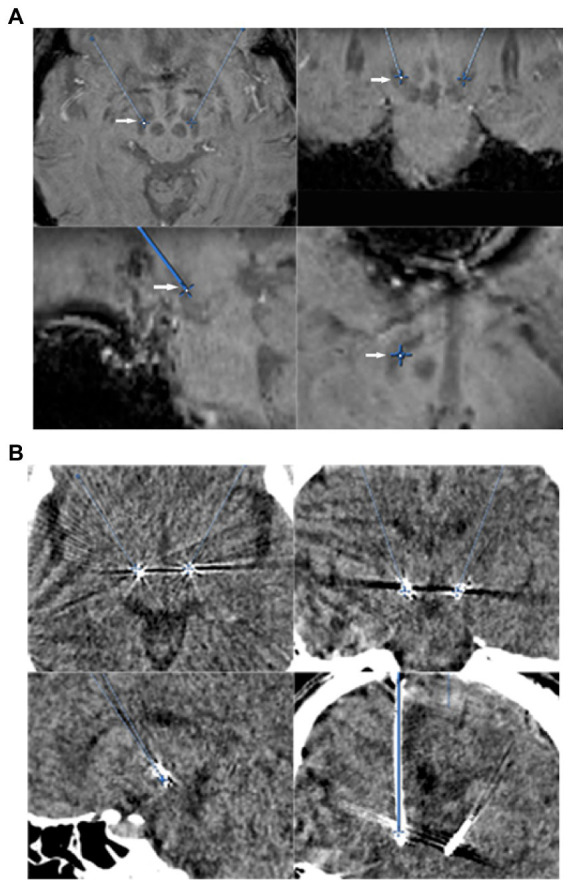
**(A)** STN direct targeting (arrows) on an SWI-sequence. Axial (upper left), coronal (upper right), and sagittal (lower left), trajectory view (lower right). **(B)** Post-electrode placement CT scans showing contact place to target, axial (upper left), coronal (upper right), and sagittal (lower left), and trajectory view (lower right).

All patients underwent a 3.0-tesla MR scan (General Electric or Siemens) before surgery. The standard settings for STN targeting comprised T2-weighted axial images at 2 mm thickness and susceptibility-weighted imaging (SWI) at 2 mm thickness. Anterior commissure (AC)–posterior commissure (PC) coordinates and midline were used as an initial guide for setting the preliminary target plan. The tentative STN target coordinates were set at about 6 mm aside from the top tangential line point at the red nuclei’s maximum diameter layer or set at about 3 mm aside from the lateral at the red nuclei’s maximum diameter layer on axial view scan (Chen et al., 2011, 2018; Figure 1A).

On the morning of surgery, the patient received the disinfection and proper lidocaine local anesthesia on the shaved scalp. A Leksell stereotactic skull frame (Elekta, Sweden) was mounted on patient’s skull, after which a brain computed tomography (CT) scan was scheduled. The patient was then sent to the operation room and secured with the head frame on a Mayfield adaptor (Lifesciences Corporation, United States) lying on the operation table.

The preoperative MR scan images and preoperative head frame CT sequences were all transferred to both the BrainLab Vector-Vision Neuro-navigation workstation (Brainlab Company, Munich, Germany) and FrameLink Software (Medtronic, Inc. United States) system independently for co-register and fusion. Preoperative STN targeting coordinates were modified and determined under direct visualization. Next, the entry point and trajectory were precisely selected with avoiding sulci and ventricles.

#### 2.2.2. Anesthetic procedure

Patients received STN-DBS. For awake group, 0.5% ropivacaine was infused throughout the incision. Then drilling skull, microelectrode recording (MER), macrostimulation, and electrode implantation were performed under local anesthesia while monitoring vital signs. For asleep group, anesthesia was induced and maintained with propofol, opioid, and muscle relaxant. Drilling skull, MER passive movement, and electrode implantation were performed under general anesthesia with a narcotrend-based assessment of anesthetic depth.

#### 2.2.3. MER procedure

The signal acquired from microelectrode tip was transferred to the micro-recording system (Leadpoint; Medtronic, United States) (Jiang et al., 2021). Those signals were magnified and displayed in the screen. Both in awake and asleep groups, passive movement tests of contralateral limbs were done and repeated for observing any movement-evoking neuronal firing changes during microelectrode penetrating toward STN (Chen et al., 2018). In awake group, the patients received macrostimulation test up to 5 V for side effects. A neurologist, neurosurgeon, and anesthesiologist analyzed signals together. A final appropriate trajectory was selected based on satisfactory signals. Intraoperative fluoroscopy by a C-arm X-ray machine was used for marking microelectrode tip location. The quadripolar electrodes (Model 3,389, Medtronic, MN, United States) were implanted into STN along the above trajectory. Further intraoperative fluoroscopy by a C-arm X-ray machine was used to accurately localize the target by adjusting the electrodes with a comparison of microelectrode tip location maker.

#### 2.2.4. Impulse generator implantation and postoperative course

After the Medtronic 3,389 electrodes were permanently implanted, both awake and asleep groups received intravenous propofol general anesthesia again. Impulse generator (IPG) was implanted in the right subclavian subcutaneous package and connected to extended electrodes on the same day. Patients were sent to neurosurgery intensive care unit (ICU) for tracheal extubation and recovery after surgery. Within 24 h after the operation, brain computed tomography (CT) scanning was performed for each patient to exclude intracranial complications and preliminarily evaluate the position of the electrodes (Figure 1B). About 1 week after surgery, postoperative 1.5 T MRI brain imaging was scanned for evaluating the final position of the electrodes by image fusion.

#### 2.2.5. Initial programming

All patients were transferred to neurologists in our team about 4 weeks after DBS surgery. Anti-parkinsonian medications were adjusted based on clinical manifestations evaluation. The drugs generally remained unchanged; however, they could be reduced if dyskinesia occurred. Initial programming was performed 1 month after DBS surgery and set with low voltages from 1.0 V to 1.5 V, keeping both frequency (130 Hz) and pulse width (60 us) constant (Picillo et al., 2016). In the meantime, follow-up and clinical observations were performed strictly following designments (Xu et al., 2018). Later, those programming parameters were adjusted and determined according to patients’ clinical assessments by our qualified neurologists.

### 2.3. Postoperative follow-up and outcome analysis

Every patient was assessed by overall clinical evaluation 1-year postoperatively. For motor function evaluation, MDS-UPDRS III scale was used to assess two preoperative motor states and four postoperative motor states. Preoperative evaluation included ON MED and OFF MED states, postoperative evaluation included OFF MED/ON STIM, OFF MED/OFF STIM, ON MED/OFF STIM, and ON MED/ON STIM states. Patients were instructed to discontinue anti-parkinsonian drugs for 12 h the day before follow-up. When the patient arrived at our follow-up center, current state was evaluated as OFF MED/ON STIM state. Then implantable pulse generator (IPG) was turned off. OFF MED/OFF STIM state was evaluated 30 min later. Next, the patient chewed equal Madopar as well as that during levodopa challenge test. ON MED/OFF STIM state was evaluated after Madopar worked effectively around 30 min later. Next, IPG was turned on. ON MED/ON STIM state was evaluated after another half-an-hour. Postoperative assessment of non-motor symptoms was consistent with that preoperative process. Levodopa equivalent daily dose (LEDD), adverse events (AEs), and stimulating parameters were also recorded and compared. In order to evaluate STN-DBS effect, changes or percentage changes from the baseline of motor (MDS-UPDRS), non-motor (NMSS, PSQI, PDSS, ESS, MMSE, MOCA, HAMD, HAMA, and BDI), quality of life (PDQ39) scales were used in the comparison between two groups.

All experimental protocols complied with the Medical Ethical Committee of The First Affiliated Hospital, Sun Yat-sen University. Signed informed consent for collecting personal medical records and videos was obtained from each participant before they entered the study. Videos were preoperatively and postoperatively recorded for each state of motor condition.

### 2.4. Statistical analysis

Statistical analyzes were performed with SPSS software, version 23.0 (SPSS Inc., IL, United States). Continuous variables were presented as mean ± standard deviation (SD). Kolmogorov–Smirnov test was used to test for normality. Normal distribution data were analyzed using independent samples t-test or paired-samples *t*-test. Nonparametric analysis was analyzed using Mann–Whitney *U* test or Wilcoxon signed-rank test. Differences between groups were expressed as simple differences or as ratios. Pearson Chi-square test, Linear-by-Linear association, and Fisher’s exact test were adopted for categorical variables. Multivariate regression and univariate regression analysis were used. A two-sided *p* < 0.05 was considered as statistically significant.

## 3. Results

### 3.1. Preoperative status of patients

Preoperative clinical characteristics of both groups are presented in Table 1. There was no significant difference in sex, age, duration of disease, LEED, and MDS-UPDRS assessments between two groups.

**Table 1:** Preoperative clinical characteristics.

**Baseline data**		**Awake group *n* = 21**	**Asleep group *n* = 25**	***p* value**
**Sex (number)**	**Male**	15	17	0.801^b^
	**Female**	6	8	
**Age (years)**		53.81 ± 8.29	57.37 ± 8.09	0.149^a^
**Duration of disease (years)**		9.05 ± 2.67	10.36 ± 3.51	0.167^a^
**MDS-UPDRS III**		50.57 ± 11.54	53.36 ± 15.95	0.508^a^
**Levodopa challenge test (%)**		60.56 ± 15.72	57.80 ± 14.32	0.572^a^
**LEDD (mg)**		931.49 ± 450.52	808.00 ± 242.57	0.269^a^

The value was presented as mean±standard deviation. ^a^This variable was analyzed using the *t*-test, ^b^These variables were analyzed using Fisher’ exact test (two-sided).

### 3.2. Stereotactic coordinates

Comparison of stereotactic coordinates, trajectory angles, MER tracts, recorded STN signal start and final sites, and effective STN depth in asleep and awake groups are presented in Table 2. The anterior commissure (AC)–posterior commissure (PC) coordinates for awake and asleep group were showing no difference. While for the left-side Y, asleep group (*Y* = –2.39 ± 0.23) showed more posterior than awake group (*Y* = –1.46 ± 0.22) compared with MCP (*p* = 0.007). Other postoperative surgical coordinates of the left-side for awake and asleep group were showing no difference. Surgical coordinates of the right-side for awake and asleep group were showing no difference. These were no difference for both lateral angle and A-P angle, mean average MER tracts comparing awake and asleep group at left and right side. The left-side and right-side recorded STN signal of awake and asleep group were showing no significant difference of STN recorded effective depth.

**Table 2:** Comparison of stereotactic coordinates, trajectory angles, MER tracts, recorded STN signal start and final sites, and effective STN depth.

**STN**	**Left-side leads**			**Right-side leads**		
	**Awake group**	**Asleep group**	***p-*value**	**Awake group**	**Asleep group**	***p-*value**
*X* (mm)	−11.12 ± 0.20	−11.62 ± 0.18	0.087^a^	11.47 ± 0.30	11.49 ± 0.24	0.892^c^
*Y* (mm)	−1.46 ± 0.22	−2.39 ± 0.23	0.007^a*^	−2.04 ± 0.32	−2.39 ± 0.30	0.356^c^
*Z* (mm)	−6.70 ± 0.32	−7.00 ± 0.30	0.494^a^	−6.54 ± 0.43	−6.6 ± 0.36	0.923^a^
Lateral angle (°)	20.20 ± 5.30	20.65 ± 4.10	0.795^a^	20.02 ± 3.66	21.03 ± 3.45	0.442^a^
A-P angle (°)	32.46 ± 6.55	32.79 ± 9.08	0.909^a^	33.21 ± 5.18	32.20 ± 8.12	0.688^a^
MER tracts	1.53 ± 1.07	1.61 ± 1.03	0.704^c^	1.24 ± 0.56	1.52 ± 1.12	0.624^c^
STN-start	−3.71 ± 1.24	−3.64 ± 1.68	0.705^c^	−4.02 ± 1.32	−3.61 ± 1.92	0.425^a^
STN-final	2.09 ± 1.15	2.33 ± 1.28	0.892^c^	1.85 ± 1.25	2.15 ±1.73	0.548^a^
Effective depth (mm)	5.79 ± 1.03	5.92 ± 0.81	0.645^c^	5.88 ± 0.83	5.76 ± 0.94	0.745^c^

a This variable was analyzed using the *t*-test, ^c^These variables were analyzed using the Mann-Whitney *U* test, *p-value <0.05.

### 3.3. MDS-UPDRS score and H-Y stage outcome

MDS-UPDRS scores were obtained in 21 patients enrolled in awake group and 25 patients in asleep group at 1-year follow-up appointment. Above all, STN-DBS treatment resulted in significant improvement in MDS-UPDRS III [its four different aspects (tremor, rigidity, akinesia, axial symptoms)] and H-Y stage in both groups. Compared with preoperative OFF MED, the improvement rate of MDS-UPDRS III at postoperative OFF MED/ON STIM state showed no significant difference (*p =* 0.530) between two groups, there was no significant change in postoperative OFF MED/OFF STIM state in both groups. Compared with preoperative ON MED, postoperative ON MED/ON STIM state showed no significant difference in both groups. Furthermore, the H-Y stage showed no difference in improvement in either group without baseline difference (Table 3).

**Table 3:** Comparison of motor outcomes.

Motor outcome	STIM state	MED-OFF							MED-ON						
		Awake group		Asleep group		Improvement			Awake group		Asleep group		Improvement		
		Baseline	1 year	Baseline	1 year	Awake group	Asleep group	*p*	Baseline	1 year	Baseline	1 year	Awake group	Asleep group	*p*
MDS-UPDRS III	off	50.57 ± 11.54	50.10 ± 12.45	53.36 ± 15.95	55.28 ± 16.12	-0.04± 0.36	-0.07 ± 0.26	0.453^c^	19.05 ± 7.22	25.71 ± 14.40	21.72 ± 8.87	29.92 ± 15.83	-0.60 ± 1.38	-0.46 ± 0.68	0.460^c^
	on		28.24 ± 10.14		26.64 ± 11.44	0.43 ± 0.21	0.47 ± 0.22	0.494^a^		15.81 ± 7.96		16.84 ± 8.83	0.10 ± 0.52	0.19 ± 0.40	0.530^c^
Tremor	off	7.38 ± 5.50	6.43 ± 4.66	7.52 ± 6.06	7.56 ± 6.37	-0.35 ± 2.12	-0.12 ± 0.88	0.605^c^	1.19 ± 1.54	1.57 ± 2.11	1.24 ± 1.48	3.08 ± 4.98	0.40 ± 0.53	-1.27 ± 3.35	0.168^c^
	on		2.86 ± 3.37		2.24 ± 3.11	0.66 ± 0.37	0.64 ± 0.48	0.846^c^		1.00 ± 1.38		0.36 ± 0.95	0.22 ± 0.75	0.63 ± 0.72	0.087^c^
Rigidity	off	11.29 ± 2.22	10.90 ± 3.51	11.12 ± 2.80	11.00 ± 2.87	0.01 ± 0.38	-0.02 ± 0.30	0.860^c^	5.95 ± 3.35	6.19 ± 3.71	6.20 ± 3.14	6.04 ± 3.81	-0.10 ± 0.81	-0.10 ± 1.00	0.945^c^
	on		6.10 ± 2.97		5.00 ± 3.63	0.46 ± 0.27	0.54 ± 0.33	0.361^a^		3.86 ± 3.10		3.36 ± 3.38	0.23 ± 0.74	0.50 ± 0.49	0.127^c^
Akinesia	off	22.90 ± 6.57	24.00 ± 7.48	23.48 ± 6.51	26.12 ± 6.50	-0.13 ± 0.53	-0.15 ± 0.29	0.871^a^	9.36 ± 3.74	13.62 ± 8.66	10.44 ± 4.59	14.24 ± 6.84	-0.66 ± 1.28	-0.47 ± 0.72	0.757^c^
	on		14.10 ± 4.83		13.60 ± 5.38	0.34 ± 0.31	0.39 ± 0.26	0.552^a^		8.14 ± 4.35		9.40 ± 4.43	0.03 ± 0.58	0.06±0.34	0.708^c^
Axial symptoms	off	9.00 ± 3.80	8.76 ± 3.94	11.24 ± 4.73	11.20 ± 5.80	-0.09 ± 0.59	-0.04 ± 0.47	0.766^c^	2.55 ± 2.22	4.33 ± 3.34	3.84 ± 2.78	5.64 ± 4.80	-1.82 ± 3.42	-0.84 ± 1.22	0.748^c^
	on		5.19 ± 2.71		5.80 ± 3.76	0.37 ± 0.36	0.45 ± 0.35	0.501^c^		2.81 ± 2.29		3.72 ± 3.16	-0.58 ± 1.61	-0.28 ± 1.35	0.739^c^
H&Y stage	off	2.90 ± 0.70	3.10 ± 0.70	3.20 ± 0.41	3.24 ± 0.72	-0.10 ± 0.27	-0.02 ± 0.21	0.982^d^	2.05 ± 0.67	2.38 ± 0.67	2.52 ± 0.51	2.48 ± 0.71	-0.28±0.48	-0.01±0.30	0.865^d^
	on		2.33 ± 0.48		2.28 ± 0.54	0.16 ± 0.24	0.28 ± 0.17	0.521^d^		2.10 ± 0.63		2.00 ± 0.65	-0.14±0.51	0.17±0.31	0.454^d^

a This variable was analyzed using the *t*-test,

c These variables were analyzed using the Mann-Whitney *U* test,

d *p* value of Covariance analysis after correction baseline.

MDS-UPDRS IB, II and IV were significantly improved in both groups. There was no significant difference in improvement in any of the groups for MDS-UPDRS IA (*p* = 0.165), MDS-UPDRS IB + II (*p* = 0.939) and MDS-UPDRS IV (*p* = 0.797).

### 3.4. Non-motor outcome

#### 3.4.1. Non-motor symptoms scale

Non-motor symptoms scale is traditionally used for evaluating non-motor symptoms of PD patients in clinic. It includes cardiovascular symptoms, sleepiness, emotion, hallucination, cognition, gastrointestinal symptom, urological symptom, sexual function, and so on. In this study, the change in NMSS scale was not significantly different between awake and asleep groups postoperatively (*p* = 0.105) (Table 4).

**Table 4:** Comparison of non-motor, quality-of-life and medication outcome.

Non-motor, quality-of-life, and medication outcome		Awake group n=21		Asleep group n=25		Improvement		
		**Baseline**	**1-year follow-up**	**Baseline**	**1-year follow-up**	**Awake group**	**Asleep group**	***p-*value**
Overall non-motor	**NMSS**	39.05 ± 19.51	32.19 ± 21.86	40.12 ± 29.65	22.64 ± 26.85	ratio, –0.13 ± 1.13	ratio, 0.04 ± 1.89	0.105^c^
Sleep evaluation	**PSQI**	9.95 ± 5.12	9.81 ± 4.43	9.76 ± 3.77	6.64 ± 4.14	ratio, –0.39 ± 1.61	ratio, 0.33 ± 0.37	0.009^c*^
	**PDSS**	107.88 ± 24.34	113.35 ± 19.85	104.79 ± 18.00	119.57 ± 19.56	ratio, 0.10 ± 0.31	ratio, 0.16 ± 0.20	0.467^a^
	**ESS**	6.00 ± 4.35	5.38 ± 4.13	8.16 ± 3.02	6.44 ± 4.49	difference, 0.62 ± 5.63	difference, 1.72 ± 5.52	0.731^c^
Cognitive function	**MMSE**	27.43 ± 2.29	27.76 ± 2.02	27.24 ± 3.10	27.08 ± 3.76	ratio, 0.01 ± 0.06	ratio, –0.01 ± 0.08	0.351^a^
	**MOCA**	24.05 ±2.73	23.95 ± 4.81	23.32 ± 5.68	24.00 ± 6.06	ratio, –0.01 ± 0.16	ratio, 0.03 ± 0.14	0.529^c^
Neuropsychological evaluation	**HAMD**	8.38 ± 7.49	10.00 ± 5.80	9.40 ± 5.41	5.32 ± 3.78	difference, –1.62 ± 8.25	difference, 4.08 ± 5.55	0.008^a*^
	**HAMA**	4.29 ± 4.10	5.71 ± 4.75	7.12 ± 4.78	3.76 ± 3.87	difference, –1.43 ± 4.53	difference, 3.36 ± 4.89	0.015^d*^
	**BDI**	12.10 ± 6.77	10.76 ± 8.16	11.52 ± 7.01	7.92 ± 9.17	difference, 1.33 ± 8.36	difference, 3.60 ± 7.87	0.453^c^
Quality of life	**PDQ39**	29.40 ± 11.70	21.17 ± 12.21	29.26 ± 12.07	21.86 ± 14.35	ratio, 0.23 ± 0.43	ratio, 0.13 ± 0.79	0.834^c^
Medication	**LEED**	931.49 ± 450.52	474.68 ± 246.13	808.00 ± 242.57	426.58 ± 180.41	ratio, 0.42 ± 0.30	ratio, 0.43 ± 0.29	0.903^c^

a This variable was analyzed using the *t*-test, ^c^These variables were analyzed using the Mann-Whitney *U* test, ^d^*p* value of Covariance analysis after correction baseline, *p-value <0.05. Improvement after DBS expressed as difference or improvement ratio (100%).

#### 3.4.2. Sleep evaluation

The postoperative improvement in PSQI score was significantly higher in asleep group (*p* = 0.009). However, there was no difference in ESS in both groups after STN-DBS treatment. No significant difference was observed in PDSS improvement in either group (Table 4).

#### 3.4.3. Neuropsychological evaluation

A comparison between two groups suggested better improvement of HAMA score (*p* = 0.015) and HAMD score (*p* = 0.008) in asleep group. There was no difference after STN-DBS treatment in BDI scores in both groups (Table 4). Multivariate analysis showed HAMA baseline score (B, 0.509; 95%Cl, 0.228–0.791; *p* = 0.001) and anesthesia methods (B,3.165; 95%Cl, 0.348–5.981; *p* = 0.029) were associated with improvement of HAMA score, and HAMD baseline score (B, 0.886; 95%Cl, 0.617–1.155; *p* < 0.001) and anesthesia methods (B, 6.346; 95%Cl, 2.477–10.215; *p* = 0.002) were associated with improvement of HAMD score. Independent variables included anesthesia method, wwwwwduration of disease, age, MDS-UPDRS baseline, left Y coordinate and HAMA baseline score or HAMD baseline score.

#### 3.4.4. Cognitive function

Mini-mental state examination and MOCA scales are usually applied for cognitive function. Our data showed no significant difference in both MMSE and MOCA scales between two groups, which suggested no postoperative cognitive functional changes (Table 4).

### 3.5. Quality of life

PDQ-39 scale consists of several items, including mobility, ADL, emotion, stigma, social support, cognition, communication, and body discomfort. The mean baseline scores showed no difference between awake and asleep group. Compared with baseline in both groups, the mean improvement percentage of PDQ-39 SI at postoperative one-year was 23 ± 43 and 13% ± 79% in awake and asleep group, presenting no difference for PDQ-39 summary index (*p =* 0.834) (Table 4).

### 3.6. Medication reduction

Medication dosage was calculated as total LEDDs according to the acknowledged conversion formula previously indicated. Medication dosage baseline showed no difference between two groups. Compared with preoperative average LEDDs, postoperative LEDDs decreased by 42% ± 30 and 43% ± 29% in awake and asleep group, respectively, showing no significant difference between two groups (*p =* 0.903) (Table 4).

### 3.7. Stimulation parameters

Multiple patterns were observed until 1 year postoperatively. In asleep group, monopolar with one contact was used in 14 patients, double monopolar in 8 patients, while the complex mode was used in 2 patients and one used interleaving mode. In awake group, monopolar with one contact was used in 12 patients, double monopolar in 5 patients, and interleaving mode in another 4 patients. The average amplitude, TEED_1sec_ (Koss et al., 2005), mean frequency and mean pulse width showed no significant difference in both groups, respectively (Table 5).

**Table 5:** Stimulation parameters in a 1-year follow-up.

**Parameter**	**Awake group**	**Asleep group**	***p*-value**
**Stimulation mode (monopolar/double monopolar/interleaving/complex)**	12/5/4/0	14/8/1/2	*p* > 0.05^e^
**onset side mean voltage (V)**	2.70 ± 0.82	2.91 ± 0.77	0.374^a^
**contralateral side mean voltage (V)**	2.59 ± 0.91	2.99 ± 0.99	0.172^a^
**onset side mean pulse width (microseconds)**	69.76 ± 10.55	63.80 ± 5.64	0.050^c^
**contralateral side mean pulse width (microseconds)**	62.86 ± 5.38	65.00 ± 9.13	0.635^c^
**mean frequency (Hz)**	123.33 ± 18.53	125.60 ± 17.70	0.674^a^
**onset side mean TEED**_**1sec**_ **(uJ)**	49.54 ± 24.51	57.48 ± 29.06	0.335^a^
**contralateral side mean TEED**_**1sec**_ **(uJ)**	43.33 ± 29.47	54.86 ± 33.68	0.162^c^

a This variable was analyzed using the *t*-test, ^c^These variables were analyzed using the Mann-Whitney *U* test, ^e^*p*=0.938 for monopolar and *p*=0.539 for double monopolar were analyzed using Pearson Chi-square test, 0.247 for interleaving was using Linear-by-Linear Association, 0.493 for complex was using Fisher’s exact test.

### 3.8. Adverse events

Adverse events (AEs) were observed in 53 patients; there was no statistical difference except incidence of hiccup was higher in asleep groups (0% for awake group and 24% for asleep group, *p* = 0.030) between awake and asleep groups (Table 6).

**Table 6:** Adverse events in a 1-year follow-up.

**Adverse events**	**Awake group *n* = 24**	**Asleep group *n* = 29**	***p*-value**
**AEs related to surgery**	2	8	0.077^b^
Hiccup	0	7	0.030^b*^
Subcutaneous seroma	0	1	1^c^
Pulmonary infection	1	0	0.453^c^
Confusion	1	0	0.453^c^
**AEs related to DBS device**	0	0	NA
**AEs related to stimulation or disease**	16	21	0.650^a^
Weight gain	6	11	0.315^a^
Dyskinesia	8	3	0.087^b^
Dysarthria	4	6	0.984^b^
Hypomania	1	6	0.174^b^
Deterioration of motor symptom	3	2	0.824^b^
Eyelid opening apraxia	2	2	1^b^
Gait disturbance(freezing/imbalance)	2	4	0.850^b^
Hallucination(visual,acoustic)	2	3	1^b^
Impulse control disorder	1	1	1^c^
Dyssomnia	3	0	0.173^b^
Dystonia	2	0	0.200^c^
Anxiety	0	1	1^c^
Apathy	0	1	1^c^
Cognitive impairment	2	1	0.866^b^
Suicide ideation	1	0	0.453^c^
Death	2	0	0.200^c^

The value was presented as a rate or composition. NA: not available. ^a^This variable was analyzed using the Pearson Chi-square test. ^b^These variables were analyzed using the Linear-by-Linear Association. ^c^These variables were analyzed using Fisher’s exact test, *p-value <0.05.

## 4. Discussion

This was a retrospective study that compared Parkinson’s patients undergoing DBS surgery under awake and asleep anesthesia state. Our results showed that PD patients’ STN-DBS postoperative symptoms were improved in both awake and asleep groups. We found no difference in MDS-UPDRS outcomes, quality-of-life, degree of medication reduction, and cognitive function between awake and asleep groups. The safety of asleep group was consistent with awake group. In addition, asleep group showed better improvement in motion and sleep evaluation than awake group. Thus, asleep STN-DBS treatment may be a good alternative option for eligible PD patients.

### 4.1. Surgical issues

Recent clinical studies have shown that STN-DBS for treating PD under GA and LA were comparable (Chen et al., 2011, 2018). Our study applied the MER and passive movement under the GA state for guiding STN electrode implantation, which could be used for STN electrode implantation as similar as reports (Collins and Wanklyn, 2002; Lin et al., 2008; Chen et al., 2018). Intraoperative fluoroscopy by a C-arm X-ray machine was applied for double-checking the final electrode position to improve the electrode implantation accuracy.

We found no difference in surgical coordinates between awake and asleep group except left-side Y value. Compared target STN surgical coordinates in two groups, posterior distance from midpoint of anterior commissure–posterior commissure plane (MCP), short as Y axis value at left-side showed more posterior in asleep group than awake group. Recent research shows that supine position and different anesthesia intraoperative blood pressure management of DBS could result in cerebrospinal fluid leaking or air inflow, which may cause unbalanced pneumoencephalus and brain shift, the difference of left-side Y maybe caused by unbalanced pneumoencephalus (Nazzaro et al., 2010; Piacentino et al., 2021). Our intraoperative STN neuronal firing (data not showed) was consistent with Chen (Chen et al., 2018). Our average MER tracts under LA and GA were 1.50 ± 0.26 and 1.60 ± 0.22, which was less than 2.05 and 2.43 reported in Chen’s study (Chen et al., 2018). McClelland’ team has shown that the depth of less than 4.5 mm might predict a poorer result, while a longer depth of MER marked STN could suggest a positive outcome after surgery (McClelland et al., 2005). For MER marked effective STN depth, our left-side depth under awake and asleep condition were 5.80 ± 0.25 mm and 5.90 ± 0.17 mm, which was longer than 4.60 ± 0.60 mm and 4.72 ± 0.68 mm of SY reported by Chen et al. (2018). A similar result was also observed in right-side depth. Our effective STN depth predicted a positive outcome for STN-DBS PD patients.

### 4.2. Motor outcome

In this study, we chose MDS-UPDRS score to evaluate motor function. MDS-UPDRS is more practical and precise compared to traditional UPDRS score (Goetz et al., 2008). We evaluated 6 states in MDS-UPDRS III, including two preoperative states and four postoperative states. The comparison of postoperative ON STIM/OFF MED state with preoperative OFF MED state was to exclude the effects of medicine and observe the effects of STN-DBS. We found significant improvements in MDS-UPDRS III, tremor, rigidity, akinesia, and axial symptoms in both groups (improvements of 43%/66%/46%/34%/37% in awake group; 47%/64%/54%/39%/45% in asleep group respectively). The biggest improvement was observed for tremor symptoms, followed by rigidity; yet, no statistical difference was observed between two groups. A previous meta-analysis found no difference in motor function outcomes in patients undergoing DBS with general and local anesthesia (Foley, 2009). Additionally, a recent clinical trial found no difference in improvement in postoperative UPDRS-III scores compared to preoperative scores between awake and asleep group (Chen et al., 2018). Our results were consistent with previous results, arguing that asleep STN-DBS made comparable motor function improvements with awake STN-DBS. There was no significant difference in OFF MED with OFF MED/OFF STIM status and ON MED with ON MED/ON STIM status, which indicated PD had no obvious progress in 1 year after an operation in both groups. No significant difference between ON MED and ON MED/OFF STIM status showed similar responsiveness to drugs.

### 4.3. Non-motor outcome

Due to complexity of non-motor symptoms, effect of DBS on non-motor symptoms (NMS) in Parkinson’s disease is unclear. NMS is prevalent throughout PD disease progression, accompanied by motor symptoms, and dominating the premotor stage (Chaudhuri and Schapira, 2009). In this study, we evaluated the NMS of PD patients *via* different clinical scales in order to reduce patients’ subjective bias. A previous study found that Chinese version of the NMSS was a comprehensive and useful measure for NMS evaluation of Chinese PD patients’ (Wang et al., 2009). This study found no significant difference in NMSS scores between awake and asleep groups. Previous studies reported improved NMSS scores for patients who underwent STN-DBS, but there was no comparative study in NMSS scores between awake and asleep DBS groups (Nazzaro et al., 2011; Wolz et al., 2012; Dafsari et al., 2016).

For sleep evaluation, three scales were applied to assess patient’s sleep in this report. ESS scale was designed to evaluate the symptoms of excessive daytime sleepiness in PD patients. Our study showed that STN-DBS surgery did not significantly improve excessive daytime sleepiness in both groups, which is consistent with Amara’s results (Amara et al., 2012). PDSS scale and PSQI scale both aimed at sleep quality assessments. PDSS mainly focused on characteristic clinical symptoms of PD patients, while PSQI focused on non-specific nocturnal symptoms. The results of Dafsari and Hjort showed that DBS could improve PDSS scores in PD patients within 3 months and 2 years after surgery. For PSQI score, Amara showed that PSQI was improved 6 months after DBS (Amara et al., 2012), while Chen reported no significant improvement at 1-year follow-up of STN-DBS (Chen et al., 2011). So far, in our center, Liu reported an advantage on post-operative PDSS in 6 months follow-up (Liu et al., 2020). In this study, there was no difference in improvement assessed by PDSS scale between two groups. Besides, asleep group showed more improvement in PSQI scale than awake group. Our results suggested that sleep quality improvement in asleep group may better than in awake group.

As for neuropsychological evaluation, Follett reported that according to BDI scores, levels of depression worsened after STN-DBS (Follett et al., 2010). Fluchere reported that BDI scores showed no significant modification at 1 year and 5 years in GA STN-DBS (Foley, 2009). Witt found a significant improvement in anxiety scales at 6-month follow-up after STN-DBS (Witt et al., 2008). Our study found greater improvement in depression and anxiety postoperatively in asleep group than in awake group, which is consistent with Lu et al. (2022).

In order to confirm what causes difference of non-motor symptoms between two groups, we used univariate and multivariate analysis, which showed that improvement in non-motor symptoms was related to anesthesia methods, this is a new discovery worth exploring. We consider improvement of sleep and mood may be caused by the long-term effects of general anesthesia. Some studies have showed that some drugs used in general anesthesia may have therapeutic effects in some psychiatric disorders (Vutskits, 2014, 2018). However, what is the underlying mechanism, how long this effect will last and whether there are other unknown factors are unclear, further long-term investigations and large-scale studies are needed.

For cognitive evaluation, overall cognition was measured by MMSE and MOCA scores. Results remained stable without an obvious change in both groups, which was in line with Brodsky’s (Foley, 2009) and Lefranc’s study (Lefranc et al., 2017). In contrast, Chen reported a significant deterioration in asleep group compared to awake group (Chen et al., 2011). Moreover, Kurtis reported a moderate decrease in verbal fluency after STN-DBS in long-term follow-up (Kurtis et al., 2017). Thus, the relation between cognition and asleep STN-DBS surgery still needs further clinical observation.

### 4.4. Quality of life

There was no significant difference in PDQ39 scores at 1 year after bilateral awake and asleep STN-DBS in our study, which is consistent with Chen’s report (Chen et al., 2018). Additionally, Brodsky found that asleep STN-DBS for PD was superior regard to speech fluency and life quality compared with awake STN-DBS in non-motor outcomes at 6 months postoperatively (Foley, 2009). Difference in different races, PDQ39 scores baseline, and follow-up time might account for the variability of results.

### 4.5. Levodopa equivalent daily dose

Our report revealed that LEDD decreased 42% in awake group and 43% in asleep group at 1-year follow-up, showing no significant difference. Nakajima (Kwon et al., 2016) and Chen (Chen et al., 2011) also found no difference in LEDD reduction in awake and asleep STN-DBS patients at 1-year follow-up. Similarly, Chen compared 14 awake and 41 asleep patients undergoing bilateral STN stimulation and observed no difference in the decrement of postoperative LEDD between both groups (Chen et al., 2018).

### 4.6. Adverse events and stimulation parameters

Ho reported that asleep DBS might lead to more treatment-induced side effects (Foley, 2009). Furthermore, Chen discovered that asleep DBS was associated with sialorrhea and dysarthria (Chen et al., 2011). However, our results showed patients were more prone to hiccups in asleep group, the symptom usually lasted for 3–10 days after surgery and disappeared without treatment. This phenomenon suggests that the occurrence of hiccup may be related to midbrain edema after operation. Stimulation parameters showed no difference between awake and asleep groups, which was in line with previously published studies (Foley, 2009; Lefranc et al., 2017; Chen et al., 2018).

### 4.7. Limitations

This study has a few limitations. First, this was a single-center retrospective study. Second, patients were not randomly selected when they were enrolled, so there may be selection bias. Patients enrolled in this research from August 2013 to Mar 2016 all received STN-DBS under local anesthesia. From Apr 2016 to Jun 2017, those enrolled patients received STN-DBS under general anesthesia. Third, the number of patients between two groups were small, some patients did not participate in postoperative follow-up due to various reasons, which may result in a statistic power decrease. Thus, multi-center, randomized, and large-scale studies still deserve further exploration.

## 5. Conclusion

Our study showed that for PD patients receiving bilateral STN-DBS, asleep DBS could improve patients’ comfort during surgery. Compared with traditional awake DBS surgery, the improvement of motor symptoms and quality of life, reduced postoperative LEDD, stereotactic coordinates, and stimulation parameters were basically equivalent. Yet, asleep group showed better improvement in terms of mood and sleep at 1-year follow-up.

## Data availability statement

The original contributions presented in the study are included in the article/supplementary material, further inquiries can be directed to the corresponding authors.

## Ethics statement

The studies involving human participants were reviewed and approved by the Medical Ethical Committee of The First Affiliated Hospital, Sun Yat-sen University. The patients/participants provided their written informed consent to participate in this study. Written informed consent was obtained from the individual(s) for the publication of any potentially identifiable images or data included in this article.

## Author contributions

LC and JL contributed to conception and design of the study. WC, LJ, QG, WX, YML, YZ, LW, CY, SX, YH, YY, JC, RX, YL, and YTL organized the database. WC and JG performed the statistical analysis. WC wrote the first draft of the manuscript. CZ, NJ, and LJ wrote sections of the manuscript. All authors contributed to manuscript revision, read, and approved the submitted version.

## Funding

This work was supported by the National Natural Science Foundation of China (grant number 82271267); Key Realm R&D Program of Guangdong Province (2018B030337001); Guangdong Provincial Key Laboratory of Diagnosis and Treatment of Major Neurological Diseases (2020B1212060017); Guangdong Provincial Clinical Research Center for Neurological Diseases (2020B1111170002); Southern China International Joint Research Center for Early Intervention and Functional Rehabilitation of Neurological Diseases (2015B050501003 and 2020A0505020004); Guangdong Provincial Engineering Center for Major Neurological Disease Treatment, Guangdong Provincial Translational Medicine Innovation Platform for Diagnosis and Treatment of Major Neurological Disease, Guangzhou Clinical Research and Translational Center for Major Neurological Diseases (201604020010).

## Conflict of interest

The authors declare that the research was conducted in the absence of any commercial or financial relationships that could be construed as a potential conflict of interest.

## Publisher’s note

All claims expressed in this article are solely those of the authors and do not necessarily represent those of their affiliated organizations, or those of the publisher, the editors and the reviewers. Any product that may be evaluated in this article, or claim that may be made by its manufacturer, is not guaranteed or endorsed by the publisher.
